# On the Mensuration of the Human Skull

**Published:** 1861-09

**Authors:** J. Aitken Meigs

**Affiliations:** Late Professor of the Institutes of Medicine in the Medical Department of Pennsylvania College, etc.


### Original Communications.

ART. I.—*On the Mensuration of the Human Skull.* By J. AITKEN MEIGS, M.D., late Professor of the Institutes of Medicine in the Medical Department of Pennsylvania College, etc.

"Omnia *mensura*, et numero, et pondere disposuisti."
*Moschus of Sidon*, Liber Sapientiæ, xi. 21.

"La mesure de la tête, loin d'être une methode surannée, oiseuse, qu'il faut proscrire, est une découverte savante, nécessaire, que nous devons conserver. Tout modèle pris d'après nature, renferme une vérité; sachons l'étudier, le comprendre et l'utiliser."
*Deschamps*, Études des Races Humaines, 99.

THE study of human cranial forms undoubtedly constitutes a very important, if not indeed the fundamental, chapter in ethnographic morphology. For the successful prosecution of this study, exact measurements of the calvaria and face are required. Metrical data of this kind are very useful in determining the physiological relations not only of the skull to the inclosed brain, but also of the different portions of the skull to each other. A thorough knowledge of these relations is absolutely essential before any successful attempt can be made at the zoological classification of the races of men. Hence the utility of devising some systematic method of measuring the cranium has been recognized by nearly all writers upon craniography, from the days of Spigel and Severin down to the present time. "Vix opus est ut copiose dicam de utilitate," writes Professor Van der Hoeven, in the recently published Catalogue* of his cranial collection, "quæ cum diligenti notatione dimensionum in diversarum gentium craniis conjuncta est. Ut normam quamdam mediam habeamus, ut certior et aptior usus sit verborum, quibus utimur in dijudicanda cranii cujusdam aut singularum etiam partium e quibus constat magnitudine, omnino requiritur ut hæ dimensiones notentur, colligantur, conferantur. Est opus tædii plenum, quod nugatorium multis forsan videtur, sed tamen ad perficiendam craniologiam comparatam pernecessarium." Professor Von Baer, of St. Petersburg, another craniologist of repute, some time ago proposed, in view of the importance of such measurements, that a convention of anthropologists should be held for the purpose of establishing a uniform system, in accordance with which these measurements might be made, and the results obtained by different observers rendered comparable with each other, and thereby enhanced very

* Catalogus Craniorum Diversarum Gentium quæ Collegit J. Van der Hoeven. Lugduni Batavorum, 1860, p. 3.

materially in value.* Professors Huschke† of Jena, and Virchow‡ of Berlin, and others, have also, in recent publications, urged the desirability of cultivating the science of craniometry.

Most of the eminent anatomists who have given their attention to the comparative mensuration of the skull have attempted to express the relation between the calvaria and face by some single measurement or method of comparison. Thus Camper relied mainly upon the *angulus facialis;*§ Daubenton, upon the *angulus occipitalis;*|| Mulder, upon the *angulus sincipitalis;*¶ Walther, upon the *angulus cranioscopus;*** Barclay, upon the *angulus basi-facialis;*†† Serres, upon the *angulus*

---

* Nachrichten über die ethnog. craniol. Sammlung zu St. Petersburg. S. 81.

† Schædel, Hirn und Seele des Menschen und der Thiere nach Alter, Geschlecht und Race, dargestellt nach neuen Methoden und Untersuchungen. Von Emil Huschke. Jena, 1854. In this admirable and elaborate work the author endeavors, with equal zeal and skill, to establish the precise relations existing between the skull, brain, and intellectual faculties.

‡ Untersuchungen über die Entwickelung des Schädelgrundes im gesunden und krankhaften Zustande, etc. Von Rudolf Virchow, Berlin, 1857.

§ Dissertation physique de M. Pierre Camper sur les Différences réelles que présentent les traits du Visage chez les Hommes de différents pays et de différents âges, etc. Autrecht, 1791.

|| Mémoire sur les Différences de la Situation du Grand Trou Occipital dans l'Homme et dans les Animaux, in Mém. de l'Acad. Royale des Sciences de Paris, 1764, p. 568.

¶ A line is dropped from the most prominent part of the frontal bone to the most prominent of the superior maxilla; this is crossed by another line passing backward and downward through the naso-frontal suture to the spheno-basilar junction. The upper angle formed near the glabella by the intersection of these two lines is the sincipital angle. (*Dissertatio de Cranio ejusque ad faciem ratione pro gradu doctoratus, submittit W. H. Crull*, Groningæ, 1810, p. 56.)

** A line drawn from the crista galli of the ethmoid bone to the external occipital spine is crossed by one connecting the most prominent point of the os frontis with the root of the nose. The "cranioscopic angle" thus formed measures the different grades of human and humanoid cranial forms, and enables us to recognize at a glance national and even individual differences. (*Kritische Darstellung der Gallschen Anatomisch-physiologischen Untersuchungen des Gehirn und Schaedelbaues.* Zurich, 1802, viii. p. 108.)

†† "Dr. Barclay, observing that the apertures of the ear and nose differ considerably in position in different animals, and that even in man the aperture of the ear is lower during infancy than in the adult, and further observing that these variations are independent of the general proportions of the skull, retained Camper's facial line, but drew the second or basial line along the under surface of the palate, continuing it forward to meet the facial line in front of the alveolar process. His reasoning, so far as it goes, is just; and his angle not only more accurate, but easily measured than Camper's, especially with the craniogoniometer invented by Dr. Leach, which indicates the span of the angle on a graduated arc." (*Outlines of Human Osteology.* By F. O. Ward. London, 1838, p. 147.)

*meta-facialis;** and Quatrefages, upon the *angulus parietalis.*† Cuvier compared the areas of the cranium and face upon a vertical antero-posterior section of the head;‡ Blumenbach suggested the *norma verticalis;*§ Owen, the *norma basialis.*|| Tiedemann determined the internal capacity of the skull by filling it with millet-seed, and then ascertaining the weight of the seed.¶ Morton adopted a similar expedient, at first using white pepper seed, and afterward resorting to No. 8 shot.** Hamilton gauged the interior of the skull with pure siliceous sand.†† My friend and correspondent, Mr. J. Barnard Davis, estimates the internal capacity with fine Calais sand;‡‡ Volkoff, with water;§§ and Dumoutier, by means of plaster, which, when hardened, is removed by sawing the cranium in two.|||| Stratton measures the skull "by marking the quantity of water which it displaces in a receiver of known dimensions. The receiver is constructed as nearly square as possible, 10 inches long, 10 inches broad, and 8 inches deep, inside. One of the sides is a plate of glass; all the other parts are of pine deal, well saturated with paint. On the plate of glass is fixed a perpendicular scale, divided into inches and tenths of an inch. The 0, or zero of the scale, is about five inches from the bottom of the receiver, inside, which is accurately filled with water up to the 0 point before the object be immersed. In taking measurements, the head or skull must be put into the water, with the top lowermost, till the surface of the water touches the articulation of the nasal and frontal bones, and enters the opening of both ears. From the given dimensions of the receiver, it will be obvious that each inch which the water rises on the scale corresponds to 100 cubic inches, (*i.e.* 10 × 10,) and each tenth to 10 cubic inches. The use of a vernier would give single inches, or even tenths of an inch, with equal accuracy, but a practiced eye will find the aid of the vernier unnecessary."¶¶

---

* Études des Races Humaines. Par W. H. Deschamps. Paris, 1857–59, p. 92.

† Comptes Rendus de l'Academie des Sciences, t. xlvi. p. 791.

‡ Leçons d'Anatomie Comparée. t. ii. pp. 167–171.

§ De Generis Humani Varietate Nativa. Edit. 3a. Gottingæ, 1795, p. 203.

|| Memoir on the Osteology of the Orang and Chimpanzee in the Zoological Transactions, i. p. 343.

¶ Philosophical Transactions of the Royal Society. London, 1836. p. 497–528.

** Crania Americana, p. 253. See also Dr. Morton's Catalogue of Skulls of Man and the Inferior Animals. Third edition, p. 7.

†† Edinburgh New Philosophical Journal, April, 1850.

‡‡ Crania Britannica, p. 11.

§§ Notice sur l'Épaisseur du Crâne Humaine, et sur l'appréciation du volume et de la configuration du cerveau. Par M. S. de Volkoff, p. 4.

|||| Ibid., p. 4, *note.*

¶¶ Contributions to the Mathematics of Phrenology. By James Stratton. Aberdeen, 1845, p. 5.

As early as 1553 the measurement of the head appears to have severely exercised the ingenuity of the famous painter, Albert Durer, who, in his great work, *De Symmetriâ Partium in Rectis Formis Humanorum Corporum*, has given such measurements in almost every view. These, however, are more artistic in their tendency and scope than scientific.

A glance at some of the outline drawings of Durer incontestibly show that the facial line and angle were not wholly unknown to him, and that Camper has rather elaborated than invented this method of cranial measurement. He even seems to have entertained more philosophical views of cephalometry than the Dutch professor, if we may judge from the following remarkable sentence, very remarkable, when we consider the time in which it was written: "Questa certo è cosa certissima, che coloro, che intenderanno la Brutezza, e deformita facilmente intendera, che cosa egli deva schivare nell' opera incominciata per bellezza, e quanto alcuno più si discostera della brutezza, tanto più si avvicinera alla bellezza." (Lib. iii. 84.)

The invention of the *lineæ cephalometricæ* of Spigel, who died in the early part of the seventeenth century, may perhaps be regarded as constituting the earliest scientific attempt at cranial measurement. Desirous of placing the old anatomical doctrine of a determinate skull-form upon the basis of a more exact normal method, Spigel devised four lines or measurements, viz.: a facial line, drawn from the most inferior point of the chin to the most superior point on the forehead; an occipital line, drawn from the crown of the head to the atlas; a frontal line, drawn from one temple to the other; and a sincipital line, drawn from the lowest part of the ear, in the region of the mastoid process, to the highest part of the sinciput. A skull in which these four lines were equal to each other, he regarded as regularly proportioned; one in which one line differed from the other three, he considered to be irregularly proportioned; when they all differed from each other, the head was of the form known to the ancients by the term φοξὸν, (sharp-pointed or conical,) a conformation supposed to be indicative of impudence.* Although these lines are evidently not sufficient for the comparative ethnography of the present day, yet it is interesting to observe, that in ascending the zoological scale, these lines approximate equality just in proportion as the head measured approaches the human form. Herder adopted another system of measurement. His *lineæ nuchales*, three in number, were drawn from the atlas as a common centre, severally to the highest point of the crown, to the most anterior point of the frontal bone, and to the most anterior point of the superior maxilla. The angles thus formed differ in different ani-

---

* De Humani Corp. fabr. (Edit. van der Linden, Amstelod. 1645, fol. p. 16.)

mals, and according to race, sex, etc.* A vertical line, drawn from the crown to the outer meatus, and a horizontal line, drawn from the incisor teeth of the superior maxilla to the most posterior point of the os occipitis, constitute the *lineæ cephalicæ* of Doornik. The horizontal diameter was divided by the vertical into an anterior and a posterior part. Skulls were compared together by the relation of these two parts to each other.† Spix proposed the five following diameters or *lineæ cephalometricæ:* a horizontal line, drawn from the lowest point of the condyloid process of the occipital bone, to the lower edge of the superior incisor alveoli; a facial line, drawn from this last point to the naso-frontal suture; a base line, drawn from this suture back to the lowest point of the condyloid process; a coronal line, through the highest point of the crown parallel to the horizontal line; and an occipital line, through the most posterior point of the occiput parallel to the facial line. The angle formed by the coronal and prolonged facial lines approaches more and more to a right angle as the skull approximates the human form. The angle formed between the base and facial lines is, in the human skull, always obtuse. The relation of skull to face is indicated by the base line. The triangle, composed of the facial, base, and horizontal lines, shows the general figure of the face; while from the coronal and occipital, together with the prolonged horizontal and facial lines, we derive some idea of the configuration of the skull.‡ Okens's *lineæ craniometricæ* consist of the Camperian facial, and horizontal lines and the occipital line of Daubenton. These lines are prolonged till they intersect, and the angles then measured.§ Ward suggests the following as an approximative method of

* Ideen zur Philosophie der Geschichte der Menscheit, 4 Buch.

† J. E. Doornik wijsgeerig natuurkundig onderzoek aangaande den oorspronglijken mensch en de oorspronglike stammen van deszelfs geslacht. Amsterdam, 1828.

‡ Cephalogenesis. Monachii, 1815.

§ Lehrbuch der Zoologie, 2 Abtheilung, S. 660. Many of these early craniometrical methods have been compiled by Wiedemann and Crull, but the reader will find the most complete account of them in the elaborate work of Drs. Pierer and Choulant, entitled "Anatomisch-Physiologisches Realwörterbuch zu umfassender Kenntniss der körperlichen und geistigen Natur des Menschen im gesunden Zustande. Herausgegeben von Dr. J. F. Pierer und Dr. L. Choulant." Vierter Band, Leipzig, 1821, pp. 519–29.*

"Es sind," writes Choulant, in commenting upon these methods, "hierzu so viel Bestimmungen dieser Art willkommen, als nur immer mit Geist und Sachkenntniss erfunden werden mögen; denn jede derselben wird neue, viellicht übersehene oder ungekannte verhältnisse im Baue des Schädels aufdecken, welche für den sorgfältigen Naturforscher von grosser Wichtigkeit sind, und zu den erfreulichsten Entdeckungen führen Können. Jeder Schädel erhält dadurch, dass er von so man-

* I am indebted to Prof. Robley Dunglison for the opportunity of consulting a copy of this work.

estimating the proportions of the skull in different races of men: "Carry a line (*a b*) from the occipital tuberosity to the root of the nose, (*i.e.* the centre of the naso-frontal suture;) meet this behind by a prolongation of the axis of the vertebral foramen, (*d f;*) in front by the line (*a c*) representing the profile of the nose. The angles *d f a* and *f a c,* thus formed, indicate the sudden bends of the deuto-vertebral column in this region. Lastly, draw the facial line (*a c*) from the root of the nose to the prominence of the upper jaw, so as to form a third angle, (*f a e.*) The difference between the angles *f a e* and *f a c* indicates the relative position of the nose and upper jaw. As these three angles, *d f a, f a c,* and *f a e,* become less obtuse, and as the difference between the second and third increases by the recession of the upper jaw, the proportions of the head improve, and its form approaches ideal perfection."* Dumoutier determines the volume and form of the cranium by three planes: one, horizontal, passing through the zygomatic arches; another, vertical, passing through the centres of the external meati; and the third, antero-posterior and also vertical, passing through the longitudinal sinus and the falx major. The intersection of these three planes gives him a transverse diameter, which he names *auditory,* and which is situated in the horizontal zygomatic plane, above the centre of the external meatus, and a vertical diameter erected upon the middle of the auditory. He also adds an antero-posterior and a transverse diameter corresponding to the middle of the vertical diameter.† The measures here proposed have led Volkoff to attempt the determination of the volume of the brain by means of an imaginary parallelopipedon circumscribing the cranium.‡ Engel divides

---

nigsachen Seiten betrachtet und vielseitig genau bestimmt wird, eine sichere Diagnose, durch die er auch für den, der ihn nicht selbst sehen kann, völlig brauchbar zu anthropologischen Forschungen wird. Möchten alle, denen grössere Schädelsammlungen zu Gebote stehen, dieselben nach den mannigsachsten, nur zum Theil hier angegebenen, Verhältnissen genau bestimmen und wenigstens diese Verhältnisse bekannt machen. Sie könnten uns, durch eine gute Beschreibung untestutzt, sehr oft statt der Abbildungen genügen. Möchten Männer, denen strenge Forschung nicht fremd ist, und, wie leider so vielen jetzt verachtlich dünkt, sich dieses Gegenstandes einmal mit besonderer Liebe annehmen. Die Naturgeschichte des menschen, in der jetzt, ausser den schätzbaren Blumenbachsen Arbeiten, lange nichts Erspriessliches geliestet worden ist, würde davon die herrlichsten Früchte geniessen, und wo gibt es ein wichtigeres Studium für den Menschen, als der mensch selbst?" Pages 529–30.

* Human Osteology, p. 149.

† Notice sur l'Épaisseur du Crâne Humaine. Par M. S. de Volkoff, p. 5. Dumoutier has invented a cephalometer, which he figures in his Atlas, (Plates 48 and 49,) but gives no description of it. See Voyage au Pole Sud et dans l'Océanie. etc. Anthropologie, Paris, 1854, pp. 254–55.

‡ Ibid., pp. 5–6.

the skull by an imaginary vertical plane passing through the median line from before backward. In this plane, which he calls the *main profile section*, lie the anterior and posterior points of the foramen magnum, the superior angle of the occipital bone, and the centres of the anterior fontanelle and nasal suture. These points are next connected by straight lines, which are respectively called the long axis of the great foramen, the chord or height of the occipital bone, the chord of the parietal bones, the vertical chord or height of the frontal bone, and the posterior facial line. An irregular pentagon is thus formed, the sides and angles of which are carefully measured.* Dr. Hueck, in his excellent thesis upon the Esthonian skull, adopts the ordinary measurements of the cranium, viz.: the length, breadth, and heighth of the skull; breadth of the forehead; distance between the external margins of the orbits; distance between the malar bones; greatest breadth of the face; vertical diameter of the orbits; length and breadth of the hard palate, etc.†

According to Carus, in estimating the form and dimensions of the head, we must remember that in the three cranial vertebræ there are positive indications of the three essential faculties of the soul, viz.: will, sentiment, and intelligence; that each of these vertebræ bears a definite relation to a certain portion of the brain; that mental tendencies and the will are clearly indicated by the posterior or occipital vertebra; vegetative life and sentiment by the intermediate vertebra; acuteness of sense and intelligence by the anterior or frontal vertebra; and that the development of the vertebral bones composing the nose, the orbits and auricular apparatus is always remarkably significant not only of the development and preponderance of the senses of smell, vision, and hearing, but also of the influence of these organs upon the psychical individuality of man. All methods of portraying and measuring heads, he thinks, therefore, should conform to the anatomical peculiarities and relations of these three regions.‡

Dr. Vimont directs his attention to the frontal bone, and "uses an ingenious comparative method to exhibit the differences" presented by this bone in the various races of men. He projects a uniform square of lines as large as the largest frontal bone, divided by cross lines into nine equal

* Untersuchungen über Schädelformen. Von Dr. Joseph Engel. Prag, 1851, p. 11.

† De Craniis Estonum Commentatio Anthropologica, etc., p. 13.

‡ Atlas der Cranioscopie, oder Abbildungen der Schædel- und Antlitzformen beruehmter, oder sonst murkwuerdiger Personen. Von Dr. Carl Gustav Carus. Leipzig, 1843. Heft I., Introduction. See also Grundzüge einer neuen und wissenschaftlich begründeter Cranioscopie, (Schædellehre.). Von Dr. C. G. Carus. Stuttgart, 1841.

panels, numbered over each frontal bone set upon its orbital edge.* Pucheran† and Maslieurat Lagémard‡ have also endeavored to find in the conformation of the frontal and superior maxillary bones characteristic distinctions between the races of men; while Sœmmerring, Berard, and Dubreuil§ have sought for ethnic peculiarities in the form and situation of the foramen magnum.

Chavee is satisfied with estimating the capacity of the frontal region by taking the mean of the four following measurements: From one external meatus to the other, over—1st, the superciliary ridges; 2d, above the frontal sinus; 3d, over the frontal protuberances; and 4th, over the anterior fontanelle.|| Dr. Gosse, of Geneva, in his classical monograph on cranial deformations, adopts the ordinary measurements of the skull. He proposes, however, a peculiar method of viewing crania, in order to determine whether they be natural or deformed. The skull, deprived of the lower jaw, is placed upon a horizontal surface in such a manner that it rests upon the incisor teeth and mastoid processes. While in this position a perpendicular line is let fall from the junction of the coronal and sagittal sutures to the base. If this perpendicular line cuts the basis cranii on a line with the external auditory meatus, the skull is normal; if it falls in advance of, or behind this canal, it is deformed either accidentally or artificially.¶

The cephalic triangle of Deschamps is both ethnological and physiological in its indications. It is constructed as follows: A vertical or facial line is first drawn from a point two centimetres above the nasal protuberance, so as to avoid the frontal sinus, and made to terminate on a level with and below the symphysis menti. From this point on the forehead a line is carried to the external occipital protuberance, measuring the occipito-frontal diameter. The third line is formed by joining the point on the occiput with that at the mental symphysis. This is the oc-

---

* Traité de Phrenologie, Planche 109. See also Crania Britannica, Decade i. p. 28.

† Considerations anatomiques sur les Formes de la Tête osseuse dans les Races Humaines. Thèse pour le Doctorat en Médecine. Par Jacques Pucheran. Paris, 1841.

‡ De l'Influence du maxillaire supérieur dans la conformation de la Face, et de ses différences dans les Races Humaines. Bull. de la Société Anatomique de Paris, 1840.

§ Études anatomiques de têtes ayant appartenu à des individus de Races Humaines diverses. Comptes Rendus des Séances de l'Academie des Sciences. Paris, 1837, t. iv. p. 575.

|| De la Pluralité des Races Humaines. Par Georges Pouchet. Paris, 1858, p. 183.

¶ Essai sur les Déformations Artificielles du Crâne. Paris, 1855, p. 7.

cipito-mental diameter of obstetricians. In the triangle formed by these three lines, there are three fixed bony points, three sides of known length, and three angles whose values are easily determined by reference to the simplest principles of geometry. Deschamps claims for each of these angles a particular significance. Thus the facial angle, comprised between the vertical and oblique lines, indicates the degree of development of the face and of the senses pertaining to vegetative life; the coronal or cerebral angle, comprised between the facial and horizontal lines, indicates more exactly than in the method of Camper, the volume of the cerebrum—the seat of intelligence; the occipital or cerebellar angle, comprised between the horizontal and oblique lines, indicates the degree of development of the cerebellum, the reputed co-ordinator of motion.*

The parietal angle of Quatrefages is formed by two lines, each drawn from the most prominent lateral points of the zygomatic arch to the coronal suture immediately above, and prolonged till they meet. Prichard, long ago, called attention to the importance of this angle as a means of distinguishing the races of men, but he demonstrated no method by which it could be measured. Quatrefages has invented an instrument by which this measurement may be successfully accomplished.†

To Drs. Scherzer and Schwarz, who were attached to the recent Austrian Circumnavigatory Expedition, we are indebted for an elaborate system of measurements, applicable to the head, trunk, and extremities of man. They record the age, weight, height, strength, color of the hair and eyes, and number of pulsations of the radial artery, and then take 31 dimensions (consisting of superficial distances, diameters, circumferences, etc.) of the head, 18 of the trunk, and 21 of the extremities.‡ By the extensive practical application of this metrical system they hope to obtain the data upon which to establish a natural classification of the races of men. In this hope they were, in a measure, countenanced by the late Baron Von Humboldt, who thought that we might at length arrive at a safer result in distinguishing and determining human races by this than by any other means.§

It is impossible within the limits of this brief historical survey of the

---

* Études des Races Humaines. Paris, 1857–59, p. 101.

† Sur l'angle pariétal et sur un goniomètre destiné à le mesurer. Par M. A. de Quatrefages. Comptes Rendus des Séances de l'Académie des Sciences, t. xlvi. p. 791.

‡ On measurements as a diagnostic means for distinguishing the Human Races. By Karl Scherzer, Ph. D., and Eduard Schwarz, M.D. On board H. I. R. M.'s Frigate "Novara," Port Jackson, 1858.

§ See a notice of the above measurements contributed by J. Barnard Davis to Silliman's American Journal for Science and Art, May, 1860, p. 332.

literature of craniometry to dwell upon all the various methods which have, from time to time, been promulgated by different craniographers. In addition to the works noticed above, the reader is referred for valuable information concerning the measurement of the skull to the writings of Burmeister,* Van der Hoeven,† Lucae,‡ Retzius,§ Zeising,|| Stahl,¶ Gratiolet,** Silbermann,†† and Segond.‡‡ In the work of Deschamps, already cited, will be found a brief account of the craniometrical methods advocated by Flourens, Garnot, Laurillard, the Abbé Frere, and others.

A careful examination of the various methods of measuring the skull, just passed in review, will show that many of them fail of meeting the requirements of the craniographer because of their really limited application. More is claimed for them than they can accomplish. Though useful in pointing out certain characteristic features, they take no account of others equally, perhaps more, important. Some of them are based upon erroneous views of the relationship of the different portions of the skull to each other. Others are simply impracticable attempts to measure a solid by taking the inclination to each other of two of its planes, or by estimating the area of a systematic section, which area, as Broc has justly remarked, may serve as the base of solids having different volumes. It is very doubtful whether any technical method heretofore invented, such as Camper's or Blumenbach's, etc., or that may yet be originated, can take

* The Comparative Anatomy and Psychology of the African Negro. By Hermann Burmeister. Translated from the German by Drs. Friedlander and Tomes. New York, 1853. This is a translation of one chapter of Burmeister's "Geologische Bilder," and embodies the results of his studies upon the Natural History of the African.

† Tijdschrift voor Nat. Geschiedenis en Physiologie, I., 1835, pp. 362–371. See also Annales des Sciences Naturelles. Sec. Série, t. viii., 1837, Zoologie, pp. 116–124.

‡ Zur organischen Formenlehre. Von Dr. J. C. G. Lucae. Frankfurt-am-Main, 1844; also, Zur Architectur des Menschenschädels nebst geometrischen Originalzeichnungen von Schädeln normaler und abnormer Form. Frankf.-a.-M., 1857.

§ British Association for the Advancement of Sciences, 1847.

|| Proportionslehre des menschlichens Körper. Leipzig, 1854.

¶ Einige Klinische Studien über Schädel-Difformitäten. Von F. K. Stahl. Zeitschrift für Psychiatrie, 1855.

** Mém. sur le développement de la forme du crâne de l'homme et sur quelques variations qu'on observe dans la marche de l'ossification de ses sutures. Gazette médicale de Paris, 1856, No. 37, p. 578.

†† Mesures naturelles du corps humain. Gaz. méd., 1856, No. 12, p. 180; 1857, No. 1, p. 16.

‡‡ Procédé de mensuration de la tête, applicable à tous les vertébres et destiné à découvrir la loi des modifications réciproques entre la face et la crâne, soit dans une même espèce, suivant l'âge, le sexe, les variétés, soit d'une espèce à une autre. Gaz. méd., 1857, No. 1, p. 21.

the place of a careful and extensive series of linear and spherical measurements, both absolute and comparative, projected from prominent and well selected points. The little reliance to be placed upon any of these methods becomes very evident when we subject them to the experimentum crucis of practical trial. In proof of this statement we have only to examine critically a few of the more celebrated craniometrical formulæ proposed and advocated by different observers.

Camper, for example, thought that "the basis on which the distinction of nations is founded may be displayed by two straight lines; one of which is to be drawn through the meatus auditorius to the base of the nose, (horizontal line,) and the other touching the prominent centre of the forehead, and falling thence on the most advancing part of the upper jawbone, (facial line,) the head being viewed in profile. In the angle produced by these two lines consist, he thought, not only the distinctions between the skulls of the several species of animals, but those also which are found to exist between different nations."

The facial line of Camper merely determines the inclination of the face as compared with the forehead; the facial angle measures the degree of prominence of the superior maxillary. Neither facial line nor angle can be relied upon as a measure of the relative size and perfection of the head and face; still less can they be regarded as the indices of intellectual power, for they furnish us with no information concerning the form and capacity of the calvaria. The relative breadth of face is entirely overlooked. The inclination of the facial line is regulated by the prominence of the upper jaw and the frontal sinuses, and not by the form of the head. To render this line a standard one for comparison of skulls, it should always be drawn between fixed points—the centre of the glabella, for example, and the most prominent point of the upper jaw between the alveoli of the two incisors. Now, if the reader will refer to the plates of Camper's work, he will perceive at once that while in some heads the glabella is the most prominent point of the frontal region, or, in other words, approaches nearest the perpendicular passing through the nasal spine, in others it is not more, and sometimes even less, prominent, than a portion of the os frontis directly above it. The reason of this is obvious. In the crania of some of the inferior races of men, as the woolly-haired, typical negro, the Caribs, etc., the flat os frontis retreats rapidly from the glabella; in the higher races—the so-called Caucasian—the brow rises almost vertically over the glabella, so that the facial line prolonged from the latter point touches the central part of the forehead in the median line. In these latter cases the facial angle records to a slight extent the prominence of the forehead (which is the bony indication of the anterior development of the cerebrum) as compared with the prominence of the upper jaw. In the former examples, however, the plane of the retreating brow cannot be brought within the inclination of the facial line, and no record can, therefore, be made of anterior cerebral development. If it be resolved, on the other hand, to abandon the glabella, and let the upper end of the facial line fall upon the *most prominent point* of the os frontis, wherever that may be, then, inasmuch as this point does not occupy the same location in different skulls, it will readily be seen that, in the attempt to include the facial and calvarial indications in the facial angle, we deprive it of all utility by rendering it no longer a method of comparative measurement.

To determine the value of this facial line, a horizontal line, as is well known, was drawn by Camper through the external auditory meatus to the base of the nose. Dr. Morton, in making his measurements with his facial goniometer, " uniformly carried this line to the nasal spine, above and between the roots of the two front incisor teeth."* Any one who is accustomed to observe crania knows very well that in different skulls it often happens that, while the distance between the meatus and the centre of the naso-frontal suture remains the same, the distance between this latter point and the nasal spine varies; in other words, the height of the face seems often to vary independently of the length of the skull. But very obviously, any variation in the position of the nasal spine must alter sensibly the facial angle. Depression of the spine will render the angle more acute, elevation of it will make the angle approach a right angle. In fact, the only fixed point in the two lines is the external meatus, which, as a point of departure in the comparative mensuration of the skull, is of the first importance.

The Camperian method entirely overlooks the breadth of the face. Crania of different races, differing completely in their ostéological characters, have the same facial angle, while others of the same race, resembling each other closely in bony characters, differ much in this angle. This was long ago shown by Blumenbach in his *Decades Craniorum*, in the case of certain negro and Polish skulls, and has been verified by almost every craniographer since his time.

The different methods employed by different observers to measure the facial angle prevent the results of their experiments from being comparable with each other. To measure this angle with unerring mathe-

---

* Crania Americana, p. 250. J. Cloquet, in order to take into account any accidental prominence of the superior maxilla and teeth below the nasal spine, causes the facial and horizontal lines to meet at the level of the upper teeth. To obtain a still more exact measurement of the extent of the face, Deschamps proposes to prolong both lines to the symphysis menti. (See Ẽtudes des Races Humaines. Methode naturelle d'Ethnologie. Par Michael-Hyacinthe Deschamps. Paris, 1857. pp. 96.)

matical precision, an accurate photographic outline of the head in a lateral view should, as I have elsewhere said,* be first obtained; upon this figure the facial and horizontal lines of Camper should next be drawn, and the angle then measured with a finely-graduated protractor. This was essentially the method employed by Camper, and quite recently, according to Deschamps, by M. Rosseau, of the Museum d'Histoire Naturelle, who accompanied the expedition of Prince Napoleon to the Polar Circle.† Morton made use of the facial goniometer, described in *Crania Americana,* an instrument not to be relied upon, since, in different but equally careful hands, it is very liable to yield different results for the same head. Cuvier and Geoffroy St. Hilaire ascertained the facial angle by the following method: the external auricular orifices are connected with a line, and upon this, as a base, is erected an isosceles triangle, the sides of which are formed by a line drawn from the external auditory meatus to the edge of the incisors. A perpendicular is then let fall from the apex to the base. Another isosceles triangle is constructed upon the same base, having for its sides the distance from the auricular orifice to the most prominent point of the forehead. The summit of this triangle is also connected with the base by a vertical line. A third triangle is next formed upon the perpendicular of the first isosceles triangle, the sides being formed by the distance from the incisors to the most projecting point of the os frontis and the perpendicular of the second triangle respectively. The angle comprised between the first two of these three lines is the facial angle.‡ To determine this angle Jacquart proposes to construct a triangle the sides of which are formed as follows: 1st, by a line drawn from the middle of the external meatus to the edge of the incisors; 2d, by a line extending from the edge of the incisors to the most prominent point of the forehead; 3d, by a line falling from this point to the external auditory orifice. The angle comprised between the first two lines gives the facial angle of Camper.§ Dr. Schnepf employs the following measurements in order to determine the facial angle: with a cephalometer he measures the distance—1st, from the point of intersection of the two depressions which limit the tragus before and above to the depression of the lower lip under the nasal spine; 2d, from the first of these two points to the naso-frontal depression; and 3d, from this latter point to the depression below the nasal spine. Knowing the dimensions

* See "Cranial Characteristics of the Races of Man," in Indigenous Races of the Earth, p. 256.

† Études des Races Humaines, p. 99.

‡ Magasin Encyclopédique, 1ère année, t. iii. p. 459.

§ Mémoire sur la Mensuration de l'Angle Facial. Comptes Rendus des Séances et Mémoires de la Société de Biologie, 1856, p. 60.

and inclination to each other of the three sides of the triangle thus formed, he calculates the value of the facial angle by means of a simple trigonometrical formula approved by the mathematician Blanchet.*

From these observations, it will be seen that the Camperian lines and angles are *per se* of but little value to the craniographer.

Blumenbach, after commenting, in his essay *De Generis Humani Varietate Nativa*, upon the method of Camper, proposed his *norma verticalis*, which consists, as is well known, in resting the cranium upon its base and looking down vertically upon the vault; thus observing how far the maxillæ project beyond the frontal arch, the direction of the jaws and cheek-bones, the proportional breadth or narrowness of the head, etc.

A little reflection will show that this method is not much more useful in determining the differential characters of skulls than the preceding. It is based, in fact, upon essentially the same erroneous hypothesis that misled both Camper and Daubenton—an hypothesis whose fallacy, though apparently not wholly unknown to Durer, was first clearly pointed out by Bell. Blumenbach merely attempted to demonstrate the fundamental idea of his predecessors—the relation of face to head proper—by viewing the head perpendicularly instead of laterally. By so doing he introduced into his plates two important features—the coronal outline and breadth of face—and thus made a decided advance in craniography. The configuration of the crown differs very much in the different races of men, and should, therefore, be always considered in the ethnographic comparison of crania. But the comparison of skulls in a lateral or profile view is equally important; for this view comprehends many important points, all of which were lost sight of by Blumenbach in consequence of his entire repudiation of the Camperian method. In comparing skulls according to the Blumenbachian system, it is obvious, as Bell has said, "that we cannot see the depth of the bones of the face, nor of the cranium, and consequently we cannot observe the proportions which the area of the bones of the face has to that of the cranium."†

The illustrious Cuvier turned his attention to this subject but only to fall into the same fundamental error. His method, which is but a modification of Doornik's incisivo-occipital line, is pronounced by Bell to be indeed, "extravagantly incorrect." He divided the bones of the head into the calvarial and facial parts. The calvarial bones inclosing the brain are the osseous representatives of intellectual and moral man; the bones of the face lodge the organs of vision, smell, and taste, and are, therefore, the representatives of sensual man. "A large cranium and a

* Mensuration de l'Angle Facial. Gazette Médicale, 1856, No. 48, p. 752.

† Essays on the Anatomy and Philosophy of Expression. By Charles Bell. 2d edit., London, 1824, pp. 151–2.

small face," he observes, "indicate a large brain, and organs of smell and taste but slightly developed; a small skull and large face, on the contrary, indicate a less voluminous brain, and more perfectly developed olfactory and gustatory organs." Cuvier appears to have thought, furthermore, that the animal is, to a great extent, what it is in virtue of two great functions especially—digestion and generation—both organic, but the one individual in its relations, and the other specific. "La nature," he says, "de chaque animal dépend en grand partie de l'énergie relative de chacune de ses fonctions; il est, pour ainsi dire, entraîné et maîtrisé par celle de ses sensations qui sont les plus fortes. * * * * La proportion respective du crâne et de la face indiquant immédiatement celle du cerveau, avec deux des principaux organes extérieurs, est donc aussi un indice du plus ou moins de perfection des facultés intérieures comparées avec les extérieures. Mais il y a une considération de plus qui ajoute à son importance comme indice: c'est que les deux sens en question sont ceux qui agissent sur les animaux avec le plus de force; ceux qui les maîtrisent le plus puissamment, à cause de l'énergie que deux des besoins les plus pressants, la faim et l'amour, communiquent à leurs impressions. Les actions auxquelles ces besoins déterminent sont aussi celles dans lesquelles il entre le plus d'aveugle fureur, le plus de brutalité, s'il est peronis de s'exprimer ainsi, lorsqu'il ne s'agit pas de l'homme."*

He next premised that the sense of hunger and the degree of development of the organ of taste were, respectively, the subjective and objective indications of the nature and power of the digestive function, and that, in like manner, the organ of smell and the sexual desire were the external and internal expressions of the reproductive power. It is somewhat singular to observe that Cuvier regarded these premises as facts, whereas they are, in reality, imperfect deductions from that great and truly philosophical principle in comparative anatomy—the correlation of parts—which he did so much to develop, and in the enunciation of which, although so wedded to his doctrine of the immutability of species, and so eminently great in calm and cautious detail, he approached nearest to Gœthe and Geoffroy Saint Hilaire, the great leaders of the transcendental school, and two of the profoundest thinkers that ever lived. Cuvier's next step was a strictly logical one, one that would have occurred to any thinking mind. He announced that the relation between the bones which support the organs of sense and those which contain the organs of mind, expresses the relation which the sensual bear to the intellectual powers of man.

So far Cuvier was correct. The steps of his argument were logically successive, and were all referable to the great principle in comparative

* Leçons d'Anatomie Comparée, 2e edit., t. ii. p. 160.

anatomy to which allusion has just been made. Beyond this his critical acumen deserted him, and he signally failed in his final conclusions. The bones of the face contain the orbits, the nasal and buccal cavities, and these lodge respectively the organs of vision, smell, and taste. Now Cuvier thought that the prominence of the ossa nasi and the magnitude of the maxilla bore a fixed relation to the degree of perfection of the organs of smell and taste. Here was his great error. In attempting to contrast the *size* of a bone with the *perfection* of the organ it supported, he was really comparing things which in their very nature are incomparable. It was the old error, in fact, handed down by Galen, and not yet wholly eradicated from the schools and the books—the error of attempting to deduce physiological laws and principles from structure alone. The perfection of an organ depends not upon its size alone, but still more upon its weight, its density, and especially its quality, its chemical and physical composition, the arrangement of its ultimate particles, etc. These are all associated elements which do not admit of separation. The attempt to separate them is followed, to a greater or less extent, by inaccurate results, as shown in the example under consideration. The nervous centre of the sense of vision is the tubercula quadrigemina, and these communicate with the globe of the eye through the optic nerves and retina, which constitute the so-called vital part of the visual apparatus. To determine the organic relations of the bony orbits to this part, we must first ascertain the structural relations existing between the optic nerves and retina on the one hand, and the aqueous and vitreous humors, crystalline lens, etc., on the other, and finally the relations of these latter parts to the bony orbits themselves. In the case of the organ of smell the same remarks apply to the olfactory ganglia and commissures, the cribriform plate of the ethmoid, the distribution of the olfactory filaments, the arrangement of the Schneiderian mucous membrane, and the prominence and form of the nasal bones. With regard to the sense of taste we must study the relations existing between the gustatory nerves, the size, form, and structure of the circumvallate and fungiform papillæ of the tongue, the tongue itself, and the maxillæ.

As early as 1809 the attention of Sir Charles Bell was directed to this subject by a remark of Dr. Wm. Gibson, late Professor of Surgery in the University of Pennsylvania, and at that time a student of medicine in Edinburgh. Sir Charles informs us that Dr. Gibson "placed before him the skull of a European and of a negro, and resting them both on the condyles of the occipital bone, it appeared that the European fell forward and the African backward."* This struck him as remarkable, when both

* "Si cranium Nigritæ," writes Dr. Gibson, in his inaugural essay presented for the Doctorate, "super tabulam collocatum fuerit, maxilla inferiore amota, super physiologists and physiognomists were describing the greater comparative size of the face as the grand peculiarity of the African head. He proceeded, therefore, to investigate the matter. He selected a number of crania and divided their great convexities into degrees or equal parts, from the nasal suture to the posterior margin of the great occipital foramen. He then poised these skulls, one after the other, upon an iron rod introduced through the foramen magnum and held perpendicularly in such a manner that while the upper end or point of the rod rested against the internal surface of the vault of the cranium in the median line, the rod passed out of the foramen exactly between the condyles of the occipital bone, that is, at the point on which the head naturally rests. Confirmatory of the observation of Prof. Gibson, Bell found that in consequence of the backward inclination of the negro skull, and the forward inclination of the European cranium, the point of the rod in the former touched the inside of the cranium several degrees nearer to the bones of the face, or more forward on the skull, than in the latter. The direction of the rod, it will be seen, coincides with a perpendicular line drawn through the skull, and bearing the same relation to the bones of the calvaria that the facial line of Camper does to the bones of the face. Hence the angle formed by these two lines is a better measure of the relation of the face to the head than the old facial angle, which, in reality, only measured the inclination of the face. "We have now," writes Bell, "an explanation of the peculiarity in the position of the negro head; the upward inclination of the face, and the falling back of the head. And here, too, we have it proved that it is an error to suppose the negro head to be remarkable in character on account of any increase in the proportion of the bones of

---

basin occipitis ita innitetur, ut maxilla superior tabulam haud attingeret. E contrario, quando caput Europæi super tabulam locatum est, antrorsum fertur, et super dentes incisores maxillæ superioris restat." (Dissertatio Physica Inauguralis de Forma Ossium Gentilitia, Edinburgi, 1809, p. 25.)

Whether this observation was independently made by Dr. Gibson I cannot say. Certainly Sœmmering observed the fact, and announced it in his celebrated work published in 1785, in the following words:—

"Vermuthlich ist dieses mit die Ursache, dass, wenn man einen Mohrenschædel ohne Unterkinnlade auf eine ebne Fläche legt, er so sehr hinten aufliegt, dass die Zähne die Fläche nicht berühren, sondern um mehr als eine Linie höher gehoben werden.

"Europäische Schädel von der gewöhnlichen Form und aus dem besten Alter neigen sich meist allemal nach vorne, und ruhen eben so gut auf den Zähnen, als hinten auf. Ich weiss nicht, ob jemand diese Beobachtung vor mir gemacht habe; obgleich dies nicht alle Mohrenschädel thun." (Ueber die Körperliche Verschiedenheit des Negers vom Europäer. Frankfurt und Mainz, 1785, p. 55, § 52.) He attributes this difference to the different position of the foramen magnum and condyloid processes of the os occipitis in the two races. (P. 18, § 14.)

the face, to the area of the cranium, for the *area of the bones of the face is in this way shown to bear a proportion to the area of the bones in the cranium, less in the negro than in the European head.*

"My next object of inquiry," continues Bell, "was to find on what the distinctive character of the negro face really depends. For to the eye the *negro face appears larger, while in fact it is proved to be smaller,* than the European, considered in relation to the cranium. I took off the lower jaw-bones from both the European and the negro skull; and then, in order to poise the skulls on the perpendicular rod, it was necessary to move them *forward* on the point of the rod; but it was necessary to shift the negro skull considerably *farther* forward than the European: the point of the rod thus indicating by its removal backward on the scale that *the lower jaw of the negro bore a greater proportion to the skull than that of the European.* The facial line was of course thrown farther backward in both skulls on taking away the jaw; but the jaw of the negro being larger than that of the European, the inclination backward was greater in the negro skull. On proceeding to take away the upper jaws, and then the whole bones of the face, I found by the index on the degrees marked on the surface of the cranium, *that the jaw-bones of the negro bore a much greater proportion to the head and the other bones of the face, than those of the European skull; and that the apparent size of the bones of the negro face proceeded only from the size and form of the jaw-bones, while the upper bones of the face, and indeed all that had not relation to the teeth and mastication, were less than those of the European skull.*"

If the reader will but compare together the sentences which I have designedly italicised, and remember at the same time the important fact (seemingly wholly lost sight of by the author under notice) that the jaw-bones constitute nearly the whole of the face, he will become aware of the remarkable manner in which Bell so effectually refutes his own conclusions; for the facial bones concerned in this ethnographic question are the ossa nasi, malares, and maxillæ superior et inferior. Of these, the two maxillæ constitute by far the greater part both by size and weight. And even the malar bones are associated with the maxillæ in giving the undue size to the negro face, since the temporal muscles concerned in the movement of the jaw in the act of mastication influence in the course of their development the size of the malar bones; and as the size of this muscle corresponds to the size and weight of the jaw to which it is attached, it must be evident that the malar bones, or at least that portion of them which enters into the formation of the zygomatic arch, must correspond in form and size to the maxillæ. To be convinced of this we have but to compare the human skull with that of an herbivorous animal on the one hand, and that of a carnivorous animal on the other; the horse and lion, or bull and tiger, or sheep and dog, etc.

In the course of his investigations, Bell found that "the angle formed betwixt the perpendicular and a line drawn from the occipital hole to the superior margin of the orbit of the eye served the most distinctly to mark the variations in the inclination of the cranium." When he poised juvenile human crania of the European race upon the rod above mentioned, he found this occipito-orbital or base line of the calvaria more elevated than in adult heads of the same race, owing to the diminished size and weight of the forehead of the former. One of the most striking peculiarities of the infantile cranium is the flatness of the frontal region. As the child advances in years, and at length arrives to manhood, this flatness gradually disappears, and at last gives place to the full, rounded arch so characteristic of the higher cranial types. In the adult negro cranium this flatness being a permanent feature argues inferiority and immaturity. We are not surprised, therefore, to learn from Bell that in the negro skull the base line, as in the case of crania of white children, was much elevated—nearly ten degrees more, in fact, than in the European adult.

In summing up his experiments Bell was forced to conclude "that the relative capacity of the cranium or brain-case to that of the face, as containing the organs of the senses, is insufficient to mark the scale of intellect, or to explain the distinctions of character in the human head; that the growth of the cranium and of its contents, with the development of the faculties of the mind, is attended with a change in the shape and capacity of the fore and upper part of the cranium, in comparison with that of its back part and base; that the perfection of the human head greatly consists in the increase of the cranium forward, in the full and capacious forehead; and that the cranium of the negro, when compared with the perfect cranium of a European, has less of capacity on the fore part."

The fallacious nature of some, at least, of the above methods of skull-measurement, and the impropriety of relying upon them exclusively in our attempts to elucidate the problems of craniography, become very apparent when we consider the number and relation of the cranial bones, the numerous points of ossification which they present, and the inequality of different parts of the head in respect to the rate and extent of the ossifying process. Indeed, in view of the almost endless variations in conformation, magnitude, etc. of skulls, it would at first sight seem almost impossible to invent any metrical system by which they could be successfully compared.

In the examination and classification of his large collection of crania, Blumenbach long ago experienced this difficulty, and expressed himself in positive terms concerning it. Singularly enough, too, that remarkable man, Bernard Palissy, the potter and physicist, writing in the latter part of the sixteenth century, says very positively, in reference to cranial measurements, "Je n'y sceu iamais trouver une mesure asseurée."*

A great difficulty exists in the want of accurately-determined points from which to commence our measurements. The sutures themselves seem to be beyond all mathematical laws, while the general structure of the head is such that apparently insignificant variations in certain points are greatly magnified in their results. In a structure seemingly so irregular as a whole, therefore, and so varied as regards those points which in the main constitute national and typical characteristics, instead of relying upon one, or even a few, many judicious comparative methods are required to elicit the truth and eliminate errors otherwise unavoidable. If all things in nature be governed, as Moschus of Sidon affirmed, by measure, number, and weight, it follows that the metrical laws which govern the development of the cranial bones must express themselves in their ultimate form, and need only a certain careful inquiry to find them out. "I feel convinced," says Professor Engel, "that even the size and form of the serrations of the sutures are not left to chance, but that they have recognizable causes. I even believe that an accurate examination of the form of the sutures will enable us to gain some information of the form-development of the skull."†

Dr. Morton, as above intimated, placed great stress upon measuring the internal capacity of the skull. Nevertheless, he did not rely upon this alone, but, with a better appreciation of the requirements of craniographical science, employed in his investigations an extended series of measurements. Messrs. Davis and Thurnam, in their valuable contribution to ethnology, the Crania Britannica, have adopted a series somewhat similar to those of Morton. But a careful examination of all these meas-

---

* "Quoy voyant, il me print envie de mesurer la teste d'un homme, pour scauoir directement ses mesures, et me sembla que la sauterelle, la reigle et le compas me seroient fort propres pour ceste affaire, mais quoy qu'il en soit, ie n'y sceu iamais trouver une mesure asseurée, parce que les folies qui estoient en la dite teste luy faisoient changer ses mesures. Adonc ie fus confus, parce que ie trouvois la dite teste tantost d'une sorte et tantost d'une autre, et combien qu'aucunes fois il y eust quelque apparence de lignes directes, ainsi que i'apprestais mes outils pour les figurer, soudain, et en un moment ie trouvois que les lignes directes s'estoient rendues obliques, dont ie fus fort estonné, voyant qu'il n'y avoit aucune ligne directe en la teste de l'homme, à cause que sa folie les faisoit toutes fleschir, et les rendoit obliques. Lors ie voulus scavoir quelles espèces de folie estoient en l'homme, qui le rendoit ainsi difforme, et mal-proportionné; mais ne le pouvant scavoir ni cognoistre par l'art de géometrie, ie m'avisoy de l'examiner par une philosophie alchimistale," etc. Œuvres, Paris, 1844, p. 93. Quoted from Pouchet's *Pluralité des Races Humaines*, pp. 185–6.

† Op. cit., p. 5.

urements convinces me that they are by no means unobjectionable. They are too few in number to comprehend the almost innumerable differentiæ which separate the various race-forms of the cranium; and they are also more absolute than comparative in their scope. Cranial measurements, to be of practical use, should be carefully taken with reference to certain fixed points, and should be both absolute and relative. Absolute measurements are necessary to demonstrate those anatomical differences between the crania of different races, which assume a great zoological significance in proportion to their constancy. By relative measurements of the head we obtain an approximate idea of the peculiar physiological character of the inclosed brain; for by such measurements we can determine, in a general manner, the proportion in volume existing between the cerebral lobes or hemispheres, the cerebellum, and the medulla oblongata—the three prominent portions of the encephalon. The craniographer should determine, therefore, both the absolute size or degree of development of the entire skull and the relative size or development of its different regions—these latter being selected with reference to the various parts or organs composing the encephalon. By such measurements, preliminary and essential as they are to the study of comparative craniography, the craniographer establishes an important anatomical foundation for the researches of the psychologist—the craniographer, in fact, becomes the cranioscopist.

In accordance with these views the following cranial measurements are suggested, in the belief that they will serve materially to facilitate the ethnographic study of the human skull.

### CRANIAL MEASUREMENTS.

### I. HEAD.

1. *Occipito-Frontal*, or *Longitudinal Diameter.*—From the glabella to the most distant point of the occiput, just above the external protuberance. As the situation of this latter point varies in different skulls, its distance from the superior angle of the os occipitis should be carefully noted. This diameter expresses approximately the length or greatest antero-posterior dimension of the cerebrum, or brain proper—the seat of the intellectual and moral faculties.*

2. *Frontal*, or *Anterior Transverse Diameter.*—Between the most

* According to Mr. Stratton, the *average* length is expressed by the distance between the point of junction of the superior angle of the os occipitis with the sagittal suture and a point on the os frontis, in the median line, midway between the coronal and naso-frontal sutures. (*Contributions to the Mathematics of Phrenology*, p. 8.)

protuberant points of the os frontis, behind and above the external angular processes. This measurement gives the breadth of the anterior lobes of the cerebrum.

3. *Depth of the Supra-orbital Plates.*—Ascertained by passing a wire through the foramen lacerum orbitale, and catching the end, which is bent like a hook, against the sharp edge of the lesser wings of the sphenoid; the point of the wire which touches the supra-orbital notch is then marked with a pen, and the distance between this point and the bent extremity accurately measured. It is obvious that this diameter indicates the depth of the anterior lobes of the cerebrum.*

4. *Frontal Altitude.*—The head is so placed that the axis of the eye coincides with the plane of the horizon; one foot of a pair of callipers is then applied to the centre of the roof of the orbit, and the other made to rest upon the os frontis vertically over the first. The distance between these two points is the mean perpendicular height of the anterior lobes.

If we multiply together the 2d, 3d, and 4th diameters, we obtain an approximate measurement of the dimensions of the frontal or anterior cerebral region in cubic inches.

5. *Bi-Temporal*, or *Middle Transverse Diameter.*—The head being adjusted as above, one foot of the callipers is placed upon the most projecting point of the squamous suture, in a vertical line over the external meatus, while the other is applied to the corresponding point on the opposite side. The distance between these two points gives the breadth of the middle lobes of the cerebrum.

6. *Parietal Altitude*, or *Height of the Middle Lobes.*—Measured upon a wire passed through the foramen ovale of the sphenoidal bone to a point on the inner face of the parietal bone, directly over this foramen.

7. *Antero-Posterior Diameter*, or *Length of the Middle Lobes.*—From the anterior edge of the bony meatus, on a line with the zygoma, to a point on the temporal surface of the great wing of the sphenoid, just posterior to its malar edge.

If we multiply together the 6th and 7th diameters and the average breadth, we get the measurement of the middle region in cubic inches.

8. *Bi-Parietal Diameter.*—Between the parietal protuberances or ossific centres.

---

* The celebrated phrenologist, George Combe, suggests a simple and expeditious method of ascertaining the length of the anterior cerebral lobes in living persons. It is briefly this: the head being held in such a position that the axis of the eye is parallel with the plane of the horizon, a perpendicular line is made to cross the most prominent point of the zygomatic arch—a point easily felt in the living head. The shortest distance between this line and the superciliary ridge of that side indicates the length of these lobes. (*Crania Americana. Appendix*, p. 279.)

9. *Posterior Transverse Diameter.*—Between the posterior inferior angles of the parietalia. These two diameters give the *breadth* of the posterior lobes of the cerebrum above and below. It will be seen that the *length* of these lobes (10) is readily obtained by subtracting the sum of the 3d and 7th diameters from the 1st.

If we add the 2d, 5th, 8th, and 9th diameters together, and divide the sum by 4, the quotient will be the *average breadth* of the cranium.

11. *Vertical Diameter*, or *Depth of Skull.*—Measured from the posterior edge of the foramen magnum to a point in the sagittal suture directly above. This measurement gives the vertical thickness of the cerebellar and posterior cerebral lobes together. The external occipital protuberance indicates very exactly the position of the tentorium which separates these two divisions of the encephalon. We may avail ourselves of this fact to obtain an approximate idea of the *height or vertical thickness of the cerebellum* (12)—the co-ordinating centre of muscular motion—in the following simple manner: Let two thin and narrow slips of brass be firmly united at their ends so as to form an exact right angle like a carpenter's square. The flat side of one of these slips should be graduated in inches and fractions of an inch. Rest the skull upon its coronal surface, and apply the brass square in such a manner that the edge of the graduated limb shall touch the occipital protuberance in the median line, while the edge of the other resting upon the base coincides with the long diameter of the foramen magnum. The reading on that part of the graduated slip in contact with the occipital protuberance is the measurement required. If now we subtract this last measurement from the 11th, we get the *thickness or perpendicular height of the posterior cerebral lobes*, (13.)

If we add together the 4th, 6th, and 11th diameters, and divide the sum by 3, we get the *average height* of the skull. A close approximation to the size of the cranium in cubic inches may be readily obtained by multiplying together the average length, height, and breadth.

14. *Occipito-Frontal Arch.*—With a graduated tape over the surface of the cranium, from the posterior margin of the foramen magnum to the centre of the naso-frontal suture.

15. *Frontal Arch.*—With the tape over the frontal bosses from one meatus to the other.

16. *Parietal Arch.*—With the tape from one meatus to the other, directly over the sagittal suture.

17. *Occipital Arch.*—With the tape over the occipital protuberance, from one meatus to the other.

18. *Horizontal Periphery.*—With the tape around the cranium, so as to touch the os frontis immediately above the superciliary ridges and most prominent point of the occipital bone.

The five preceding measurements enable us to compare different skulls together, in relation to their peripheral development, with more successful results than linear measurements alone.

19. *Meato-Frontal Diameter.*—With a pair of dividers, from the meatus to the frontal boss or centre of ossification.

20. *Meato-Parietal Diameter.*—From the meatus in a direct line to the parietal centre of ossification.

21. *Meato-Occipital Diameter.*—From the meatus to the point above the occipital protuberance, indicated in the 1st measurement.

### II. HEAD AND FACE.

22. *Meato-Malar Diameter.*—From the meatus to the suture joining the malar and superior maxillary bones.

23. *Meato-Alveolar Diameter.*—From the meatus to the inferior edge of the superior alveolus in the median line.

24. *Meato-Mental Diameter.*—From the meatus to the symphysis menti.

The importance of the meatus, as a fixed and definite starting-point in the mensuration of the skull, is very evident from the above measurements. It constitutes an excellent landmark for the craniometrician, as is shown in the six diameters immediately preceding. These diameters, originating in a common centre, are strictly comparative, the three meato-calvarial or intellectual being contrasted with the three meato-facial or animal.

### III. FACE.

25. *Naso-Alveolar Diameter.*—From the nasal suture to the alveolus of the superior maxillary.

26. *Naso-Mental Diameter*, or *Length of Face.*—From the nasal suture to the point of the chin.

27. *Bi-Zygomatic Diameter*, or *Breadth of Face.*—The distance in a right line between the most prominent points of the zygomæ.

28. *Depth of the Temporo-zygomatic Fossa.*—To be taken on a level with the upper margin of the zygomatic arch.

29. *Heighth*

30. *Breadth*

31. *Direction of the Transverse Axis*

32. *Shape*

} *of the anterior opening of the orbit.*

33. *Inter-Orbital Diameter*, or *Breadth of Nose at the Root.*—From one internal angular process of the os frontis to the other.

34. *Distance between the external angular processes.*

35. *Sub-Orbital Diameter*, or *Breadth of the Superior Maxilla.*—

From the inferior point of the maxillo-malar line of junction of one side, to the corresponding point on the other.

36. *Length of Nose.*—From the nasal suture to the spinous process of the superior maxilla.

37. *Breadth of the Nasal Orifice* at its widest part.

38. *Circumference of the Upper Jaw.*—With a graduated tape, over the alveolar margin of the upper jaw from one meatus to the other.

39. *Circumference of the Lower Jaw.*—With the tape from one meatus to the other, over the point of the chin.

### IV. BASE.

40. *Inter-Auricular Diameter*, or *Breadth of the Base.*—Distance in a right line between the bony meati.

41. *Length* } *of the hard palate.*

42. *Depth* } *of the hard palate.*

43. *Breadth* } *of the hard palate.*

44. *Shape of the Superior Alveolar Arch.*

45. *Position of the Foramen Magnum.*—Determined by two measurements. *a.* From the anterior margin of the foramen to the incisor alveoli. *b.* From the posterior margin to the occiput, measured by means of the brass square above mentioned.

46. *Antero-Posterior* } *Diameters of the Foramen Magnum.*
47. *Transverse* } *Diameters of the Foramen Magnum.*

48. *Shape of the Foramen Magnum.*

